# THE IMPACT OF COVID-19 ON AVOIDANT COPING STRATEGIES AND PERCEIVED STRESS: INSIGHTS FROM THE HANDLS SLEEP STUDY

**DOI:** 10.1093/geroni/igae098.0886

**Published:** 2024-12-31

**Authors:** Junyan Tian, Alexa Allan, Lena Simon, Lesley Ross, May Beydoun, Alan Zonderman, Michele Evans, Alyssa Gamaldo

**Affiliations:** The Pennsylvania State University, Elgin, Illinois, United States; The Pennsylvania State University, Elgin, Illinois, United States; Clemson University, Clemson, South Carolina, United States; National Institute on Aging, Baltimore, Maryland, United States; National Institute on Aging, Baltimore, Maryland, United States; National Institute on Aging, Baltimore, Maryland, United States; National Institute on Aging, Baltimore, Maryland, United States; Clemson University, Clemson, South Carolina, United States

## Abstract

Coping strategies are usually defined as both effective and ineffective methods to reduce the impact of stressful life events. Avoidant coping, characterized by passive strategies, is more frequently used as people age. However, it is correlated with a higher level of perceived stress and more depressive symptoms. This study used a subsample of the Healthy Aging in Neighborhood of Diversity Across the Life Span Sleep Study (HANDLSleep) to examine the impact of the COVID-19 pandemic on the relationship between avoidant-focused coping and perceived stress. Our participants included 86 middle-aged and older adults (52.33% Black adults, age range: 46-82). The Avoidant Brief COPE scale measured how frequently they utilized avoidant coping strategies, such as substance use and denial, to disengage from stressful events. Results of the independent sample t-test indicated that individuals who tested positive for COVID-19 had lower levels of avoidant coping than those without infection, t(84) = -2.79, p <.05. Furthermore, regression analyses revealed a significant interaction effect between COVID-19 infection and perceived stress on avoidance coping, even after controlling for age, education, race and income. Specifically, only individuals who did not test positive for COVID-19 showed a correlation between higher levels of perceived stress and increased utilization of avoidant coping, b = -.02, t(76) = 2.54, p <.05. The simple slope for those with COVID-19 infection was not significant. These results highlight the significant social impact of COVID-19 infection on older adults’ coping strategy utilization when dealing with stress.

